# Coarse-Grained Models of RNA Nanotubes for Large Time Scale Studies in Biomedical Applications

**DOI:** 10.3390/biomedicines8070195

**Published:** 2020-07-06

**Authors:** Shyam Badu, Sanjay Prabhakar, Roderick Melnik

**Affiliations:** 1 MS2Discovery Interdisciplinary Research Institute, M2Net Lab, Wilfrid Laurier University, 75 University Avenue, Waterloo, ON N3L 3V6, Canada; nanommta@gmail.com (S.P.); rmelnik@wlu.ca (R.M.); 2 BCAM—Basque Center for Applied Mathematics, Alameda de Mazarredo 14, E-48009 Bilbao, Spain

**Keywords:** RNA nanotube, long-time simulation, molecular dynamics, biomedicine, drug delivery, coarse-grained model, Boltzmann inversion, nanoscale biological system

## Abstract

In order to describe the physical properties of large time scale biological systems, coarse-grained models play an increasingly important role. In this paper we develop Coarse-Grained (CG) models for RNA nanotubes and then, by using Molecular Dynamics (MD) simulation, we study their physical properties. Our exemplifications include RNA nanotubes of 40 nm long, equivalent to 10 RNA nanorings connected in series. The developed methodology is based on a coarse-grained representation of RNA nanotubes, where each coarse bead represents a group of atoms. By decreasing computation cost, this allows us to make computations feasible for realistic structures of interest. In particular, for the developed coarse-grained models with three bead approximations, we calculate the histograms for the bond angles and the dihedral angles. From the dihedral angle histograms, we analyze the characteristics of the links used to build the nanotubes. Furthermore, we also calculate the bead distances along the chains of RNA strands in the nanoclusters. The variations in these features with the size of the nanotube are discussed in detail. Finally, we present the results on the calculation of the root mean square deviations for a developed RNA nanotube to demonstrate the equilibration of the systems for drug delivery and other biomedical applications such as medical imaging and tissue engineering.

## 1. Introduction

The accurate modeling of the complex biomolecules like ribonucleic acid (RNA) nanoclusters is important for many biological processes taking place in the human body, as well as for their applications such as drug delivery. The influence of the dynamic behavior of RNA on the cellular processes of modifying genetic circuits within functionally productive pathways is rather complex [1,2,3]. With the rapid progress in the development of computational techniques and feasibility of computational resources, theoretical studies of the biomolecular systems have become more accessible. Understanding the physical properties and characteristics of complex biomolecules, such as RNA nanoclusters, is central to the rational design of new tools in biomaterials sciences and nanomedicine. Theoretical modeling and simulations provide complementary approaches for experimental studies. Moreover, the use of multiscale approaches in such cases is practically unavoidable since adsorption events span a wide range of time- and length- scales. However, it is still impossible to perform all-atom Molecular Dynamics (MD) simulation on complex biological systems, such as RNA nanotubes, for sufficiently large time scales of interest. To overcome this problem, the Coarse-Grained (CG) models are designed to perform MD simulations for large time scales in order to realistically describe the physical characteristics of these biological systems [4,5,6,7,8,9,10,11,12,13,14,15,16,17,18,19,20,21]. We note that up until now, the coarse-grained modeling of only relatively small DNA systems has been performed by using the Martini force field where the six to seven CG interaction sites are taken into account [22,23], and a similar progress has been made for RNA systems [24,25].

Current and potential applications of RNA nanotubes drive the research envelope in the development of novel CG models further. The long enough RNA nanotubes (see, e.g., [26] and references therein) are compatible to carry out drugs to damaged cells in a multitude of situations (e.g., cancer cells, cells affected by infectious diseases or viruses). For example, to deliver drugs to the human body, one could pack the (e.g., cancer) therapy reagent, like siRNA, inside the tube, and then insert it into the body with proper experimental techniques so that it will release the drugs at the necessary location. The RNA systems can be used as one of the best vehicles for drug delivery because it is compatible with the human body. Hence, the development and detailed analysis of all-atom molecular dynamics simulations and coarse-grained models of RNA nanotubes become imperative [8,24,25,27,28,29,30,31,32,33,34,35]. This is also important in the light of existing challenges in the development of RNA therapeutic methodologies, including the delivery to target cells and stability issues in blood/tissues which are subject of intensive discussions in the literature [36,37,38].

In this paper, by using coarse-grained molecular dynamics simulations of RNA nanotubes, which have not been reported anywhere, we systematically study the mechanical properties of RNA nanotubes (nanorings connected in series). Long enough RNA nanotubes, as considered in this paper, have attracted substantial interest at the practical level for drug delivery, along with other biomedical applications [34,39]. By using MD simulations based on coarse-grained models, we study the structural and physical properties of such nanoscale biological systems, which provided such characteristics as radial distributions and histograms for the bond and dihedral angles. The methodology developed here is especially useful for coarse-grained modeling of biomolecules, such as RNA nanotubes, that possess very large numbers of nucleotides in the nucleic acid. Prior studies were performed on such structures as 16-RNA molecules and 10-RNA protein complexes and some others by using generic sets of coarse-grained model parameters, with details given in [40,41,42], but not on RNA nanotubes.

## 2. Background of CG Model

In the CG modeling, when the system reaches its minimum energy equilibrium, a number of characteristics are responsible for the determination of thermodynamical properties. Among them, an important role is played by the sum of pseudo atoms, as well as the effective energy function, $U_{CG}$, as defined by Equation (1). On the other hand, for the coarse-grained model obtained from atomistic all-atom simulations, the Boltzmann inversion method has been used to fit the CG model parameters, as shown, for example, in reference [43]. We have focused our study on achieving coarse-grained model structures of RNA nanotubes from the structure of all-atom MD simulations. In this technique, the coarse-grained potential is calculated from the pairwise potential as explained in details in references [44,45]:

(1) $$U_{CG}\left(R\right)=\sum_{i<j}u_{CG}\left(R_{ij}\right),$$

where R is the vector with dimension of 3$N_{A}$ and $u_{CG}\left(R_{ij}\right)$ is the pair wise potential. This method is based on the uniqueness theorem which states that for a given pair of radial distribution functions (RDFs) there exists a pairwise potential [46]. The Boltzmann inversion method is carried out by the following six steps:

1. Perform the all-atom MD simulation.
2. Convert the all-atom simulation trajectory into the corresponding coarse-grained model and then calculate the radial distribution function for this CG pseudoatom.
3. Using these pseudoatomic radial distribution functions, calculate the pairwise potential for each pair of the CG pseudoatoms using the following Equation (2):
  (2) $$u_{CG}\left(R\right)=-k_{B}Tln\left(g_{A}\left(R\right)\right),$$
  where *T* is the temperature and $k_{B}$ is the Boltzmann constant.
4. Then perform the NVT MD simulation for the CG system using the computed pairwise potentials and determine the radial distribution function for the pseudo atoms in a CG ensemble.
5. Now, the potential can be updated by using the following relationship (3):
  (3) $$u_{CG}\left(R\right)=u_{CG}\left(R\right)-k_{B}Tln\left(g_{R}/g_{CG}\right),$$
  where $g_{R}$ is the pairwise distribution function for the all-atom MD simulation and $g_{CG}\left(R\right)$ is the pairwise potential calculated for the pseudoatomic MD simulation in the CG approximation.
6. Finally, the CG simulation is repeated as is done in step 4 until the value of $u_{CG}$ converges.

We use the above procedure in the next section for parameter fitting to obtain the structures of RNA nanotubes for the subsequent CG modeling.

## 3. Computational Details

All-atom molecular dynamics simulations have been performed by using the CHARMM force field implemented in the NAMD MD simulation package [47,48,49]. The classical equations of motion of a molecular system in the MD simulation are solved by their time-dependent integration. The potential of the system used during the molecular dynamics simulation with CHARMM force field can be expressed by Equation (4) as follow:

(4) $$\begin{array}{cc} V_{total}= & \sum_{bond}K_{b}{\left(r-r_{0}\right)}^{2}+\sum_{angle}K_{\theta }{\left(\theta -\theta_{0}\right)}^{2}\\ & +\sum_{dihedral}K_{\phi }\left(1+cos\left(n\phi -\gamma \right)\right)\\ & +\sum_{Hbond}\left(\frac{C_{ij}}{r_{ij}^{12}}-\frac{D_{ij}}{r_{ij}^{10}}\right)\\ & +\sum_{Vanderwaals}\left(\frac{A_{ij}}{r_{ij}^{12}}-\frac{B_{ij}}{r_{ij}^{10}}\right)+\sum \frac{q_{ij}}{\epsilon r_{ij}}, \end{array}$$

where the first term corresponds to bonds, second corresponds to angle parameters and so on as indicated in the Equation (4) defining the potential of the system. To get structural properties of the RNA nanotube with a coarse-grained model, we first perform all-atom MD simulations of the RNA-nanotube, which is solvated in a salty water box. The size of the box has been taken in such a way that the wall of the water box is at a distance larger than the cut-off radius. In order to make the system neutral, we have added 594, 924, 1254, and 1584 ${}^{23}Na^{+}$ for the two-ring, three-ring, four-ring, and five-ring nanotubes, respectively. Furthermore, to make the solution equivalent to the physiological solution, we have followed [50] and added an extra ${}^{23}Na^{+}$ and ${}^{35}Cl^{-}$ ions. This system was simulated at constant temperature and pressure. The temperature in the system has been controlled by using the Langevin method with damping η = 5 ps${}^{-1}$. Once the equilibrium positions for all-atom MD simulations are reached, we generate the structures of RNA nanotubes with coarse-graining models by using tcl scripting in the Visual Molecular Dynamics (VMD) program and then add the bonds between base pairs in such a way that there exist three bonds between each base pairs. For a 10-RNA nanotube, all-atom MD simulations were performed in a vacuum and then we captured the RNA nanotube structure with the corresponding coarse-grained model.

To model RNA nanotubes, we have used the RNAIi/RNAIIi as building blocks and self-assembled them to form RNA nanorings, which are later connected by links to form the nanotubes. This is done in a way analogous to references [27,51] where details of the nanoring used here can also be found. In particular, each of the nanorings consists of the six helical building blocks of RNAIi/RNAIIi complex. For the coarse-grained model of RNA nanotubes, we have used the VMD and topotool to connect the bonds between the fragments. The structure of the building blocks for the large RNA nanoclusters, i.e., RNAIi/RNAIIi, have been taken from the protein data bank with the pdb code (2bj2.pdb) [52]. The assembling of fragments of atoms for coarse-grained model structures has been done via tcl scripting in VMD. Once the nanotube model is constructed, we optimize these RNA structures by using the NAMD software [27,28,53]. In the coarse-grained modeling, a particular number of atoms are taken as a single interaction site, also known as a bead. For a nucleic acid system, one nucleotide is taken as one to three beads. For higher accuracy, one needs to choose a larger number of beads for a single nucleotide. In our case, we have taken three beads per nucleotide in such a way that the phosphate group, the sugar ring, and the nucleobase are approximated as the first, second, and third bead respectively. The masses of the beads are 109 amu for the phosphate group, 120 amu for the sugar ring, and 92.5 amu for the nucleobase. The centers of mass for these beads are taken at the phosphorous of the phosphate group, the C4 of the sugar ring, and the N1 (for pyrimidine) or N9 (for purine) of the nucleobase. As in [28], in our three-bead case, two types of beads are placed on the P atoms and C4’ carbons.

In order to fit the CG parameters for the development of model structures, we use the radial distribution functions, histograms of several bonds, angles, and dihedral angles from the all-atom MD simulations. During the calculation, the total energy of the system is coming from three different contributions. Out of these three contributions, the first one is the contribution from the chain connectivity that includes bonds, angles, and dihedral angles. The second contribution is coming from the base-pairing which comes from the hydrogen bonding between electronegative atoms and the hydrogen (electropositive) atom of two adjacent nucleobases of the RNA. Specifically, the hydrogen bonding comes from the base pairs in such a way that there is an attraction between two kinds of the base pairs, such as the cytosine pairs with guanine and the adenine pairs with the thymine. For example, in the base pairing of guanine and cytosine, there is a hydrogen bond among O6 of guanine with the hydrogen at N2 of cytosine, hydrogen at N1 of guanine with the N3 of cytosine and hydrogen at N2 of guanine with the O2 of cytosine. The last and the third contributions in the energy of the CG model comes from non-bonding interactions such as van der Waals interactions. The effective potential of mean force is calculated from the probability distribution function and the parameters are fitted by using the Boltzmann inversion method as described in Section 2 [28]. During the fitting of the parameters, following the ideas of our earlier works on RNA nanorings and more complex structures, the energy contributions from the different types of interactions in the chain are taken in such a way that they follow a certain order in terms of the strength of the interactions [19,20,21,27,28,39,50,53].

## 4. Results and Discussion

**Figure 1:**
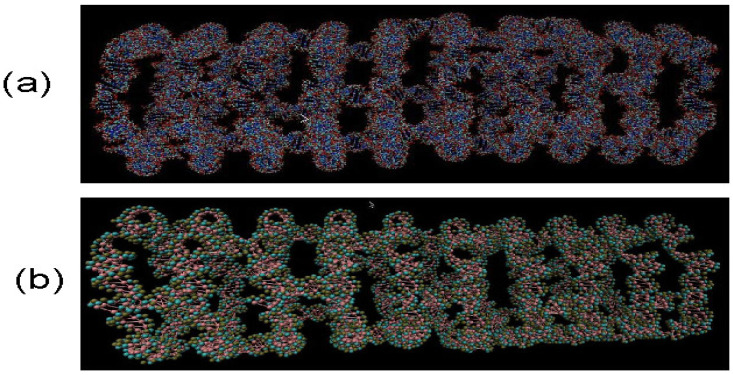
RNA nanotube made from 10 nanorings for all-atom Visual Molecular Dynamics (VMD) simulations in (**a**) and for coarse-grained VMD simulations under 3 bead approximations in (**b**). Evidently, in coarse-grained model in (**b**), the number of atoms are significantly reduced, which facilitates the calculations for analysing physical properties of RNA nanotubes.

Using molecular dynamics simulations we modeled a range of different RNA nanotubes of various sizes. One of our main results, as shown in Figure 1, is for the structures of 10-ring RNA nanotubes, obtained by performing all-atom and coarse-grained MD simulations. In modeling these 10-ring RNA nanotubes, we used the procedure described earlier. In particular, starting with the RNAi/RNAii as building blocks, they were self-assembled to form the RNA nanorings. These nanorings were connected by links to form the nanotubes [27,28,53]. As can be seen in Figure 1b for the structure of coarse-grained model simulation based on the 40nm RNA nanotube, the number of interacting sites was much smaller than for the case of all-atom VMD generated structures of the RNA nanotube. This will facilitate MD simulations aimed at the investigation of the physical properties of RNA nanoclusters based on the developed coarse-grained method [19,21]. Among others, this includes radial distribution functions, as well as histograms for the bond angles and the dihedral angles.

**Figure 2:**
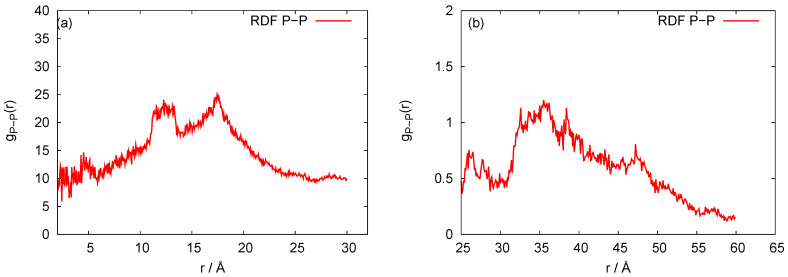
Behaviors of the P-P radial distribution functions for the five rings RNA nanotube with (**a**) all-atom Molecular Dynamics (MD) simulation and (**b**) coarse-grained approximation.

**Figure 3:**
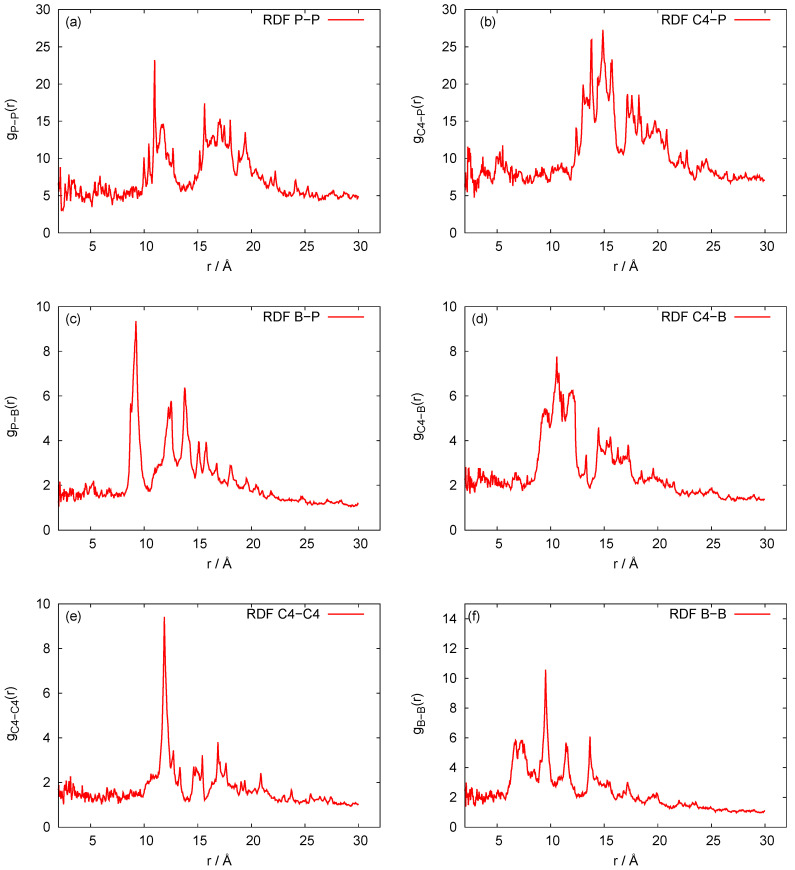
Behaviors of the radial distribution functions for the 10-ring RNA nanotube modelled with coarse-grained approximations. (**a**–**f**) present the following characteristics of this RNA nanotube: the radial distribution functions for P-P, C4-P, B-P, C4-B, C4-C4, and B-B atoms, respectively.

Our three-bead approximation is a development of our earlier model for the nanoring [28], where we adopted a representation with three beads per nucleotide that corresponds to the (P)hosphate, (S)ugar and nucleic (B)ase, respectively. It is a natural choice for nucleic acids. In Figure 2, we present the P-P radial distribution function for the RNA nanotube with five rings and the RDFs of the corresponding CG model. Here all-atom RDFs are taken from the trajectories that were calculated with representative statistical ensembles for relatively short simulation times, whereas for the CG model the simulation was performed for the 200 ns. The natures of the P-P RDF plots obtained with these two models were quite different because different numbers of atoms were assembled in the cases of all-atom and coarse-grained MD simulations of the nanotubes. In Figure 3, we present the radial distribution function of 10-ring RNA nanotube under CG model approximations. More specifically, in Figure 3a–f, we present the following characteristics of this RNA nanotube: the radial distribution functions for P-P, C4-P, B-P, C4-B, C4-C4, and B-B atoms, respectively. The peaks in RDF plots provided the distance between the considered beads used to produce the coarse-grained model structure of the RNA nanotube. As expected, such peaks were highest for P-P and C4-P atoms. The approximate distances observed in these plots were also used to self-assemble the RNA nanorings to build RNA nanotube structures of large sizes.

**Figure 4:**
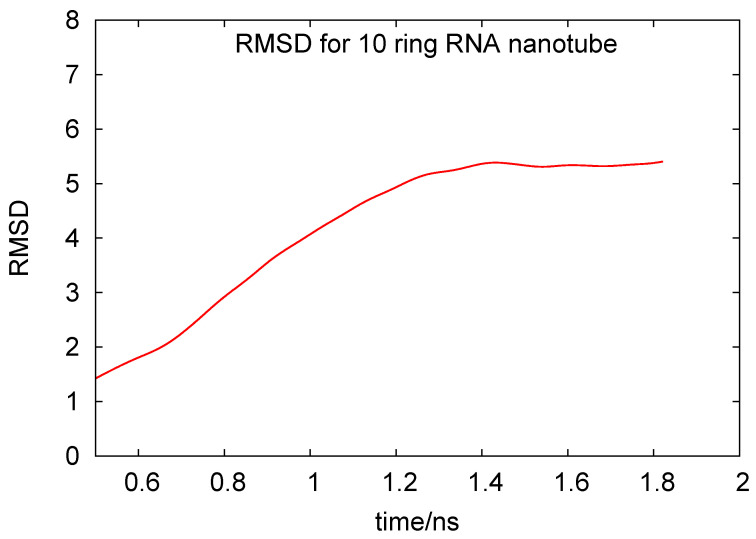
Root mean square deviation for the 10-ring RNA nanotube under coarse-grained model approximations.

**Figure 5:**
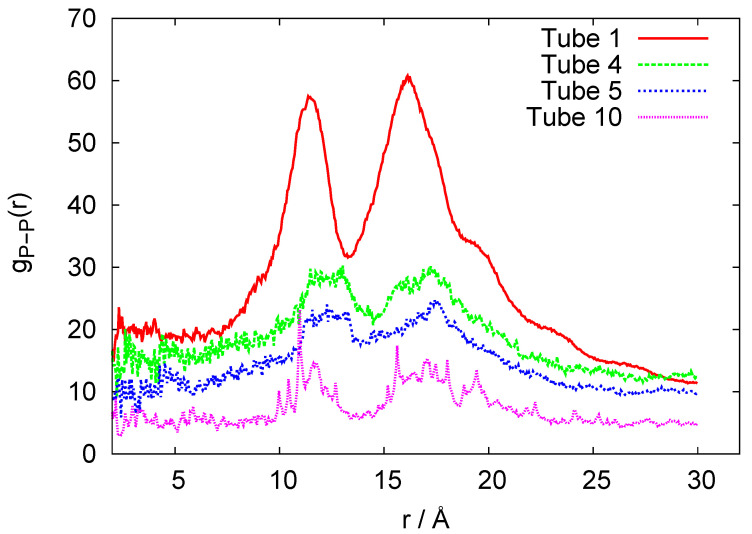
P-P Radial distribution functions for the coarse-grained models of 1-, 4-, 5-, and 10-ring RNA nanotube.

In Figure 4, we plot the root mean square deviation (RMSD) vs. time, given in units of ns, for the 10-ring RNA nanotube. As mentioned earlier, the length of such a nanotube was 40 nm. From the RMSD plot of this nanotube, we can see that the value of RMSD increased at the beginning of the simulation and then reached a constant value, indicating that the system reached an equilibrium. In Figure 5, we have presented the results for the phosphorous-phosphorous radial distribution functions of 1, 4, 5, and 10 ring RNA nanotubes. Here we see that the intensity of the radial distribution functions decreased with the increasing number of RNA nanorings in the nanotube, which also indicates that the nanotube may be modeled by discrete-to-continuum type models recently developed by our group [34,54,55].

**Figure 6:**
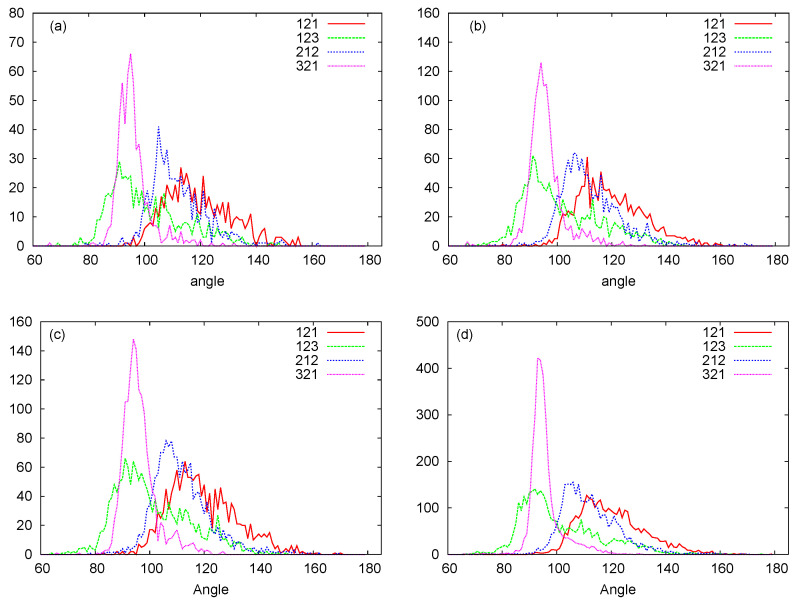
PSP, SPS bond angle histograms for the (**a**) two-ring nanotube (**b**) four-ring nanotube (**c**) five-ring nanotube (**d**) 10-ring nanotubes. (Here in the Figure 1-Phosphate (P), 2-Sugar Ring (S), and 3-Nucleobase (N).)

**Figure 7:**
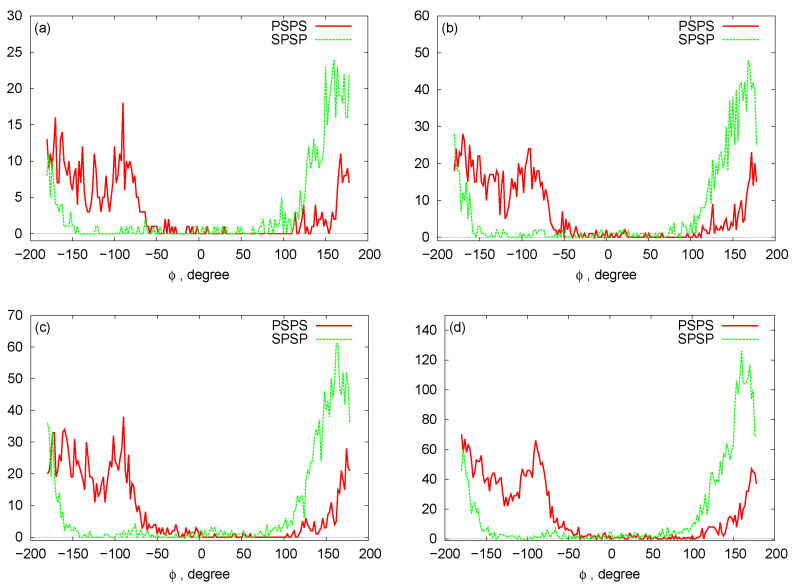
Histograms for the dihedral angles: (**a**) two-ring nanotube (**b**) four-ring nanotube (**c**) five-ring nanotube (**d**) 10-ring nanotubes.

In Figure 6, we plot histograms for the bond angles between PSP and SPS beads for the RNA nanotubes of different sizes. Evidently, the bead angles for PSP and SPS were different for different sets of beads. In particular, the highest peak was progressively increasing when we increased the number of rings in the nanotube. Furthermore, by using the three-bead approximation, we also plot the histograms for the dihedral angles for different sizes of the nanotubes in Figure 7. From these histograms, we see that the peaks for PSPS dihedral angles were found at around −100 degree for all RNA nanotubes. On the other hand, for the SPSP dihedral angles, the peaks were found to be around 150 degrees. The histogram peaks at different angles for PSPS and SPSP dihedral angles described different backbone configurations of the RNA nanotubes. As expected, the highest peak increased with an increase in the number of rings in the nanotube.

**Figure 8:**
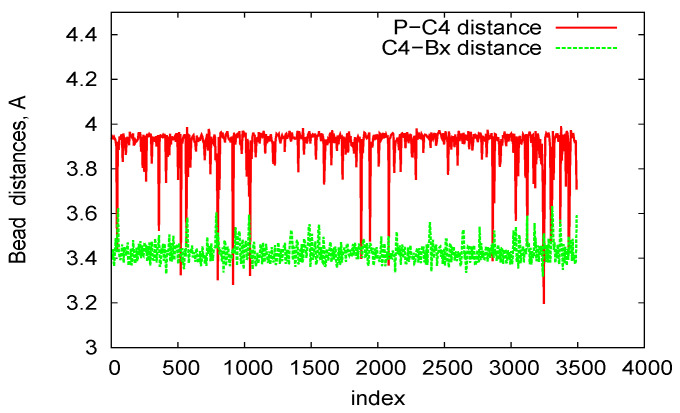
P-C4 and C4-Bx bond length in the three-bead Coarse-Grained (CG) model for the two-ring RNA nanotube.

**Figure 9:**
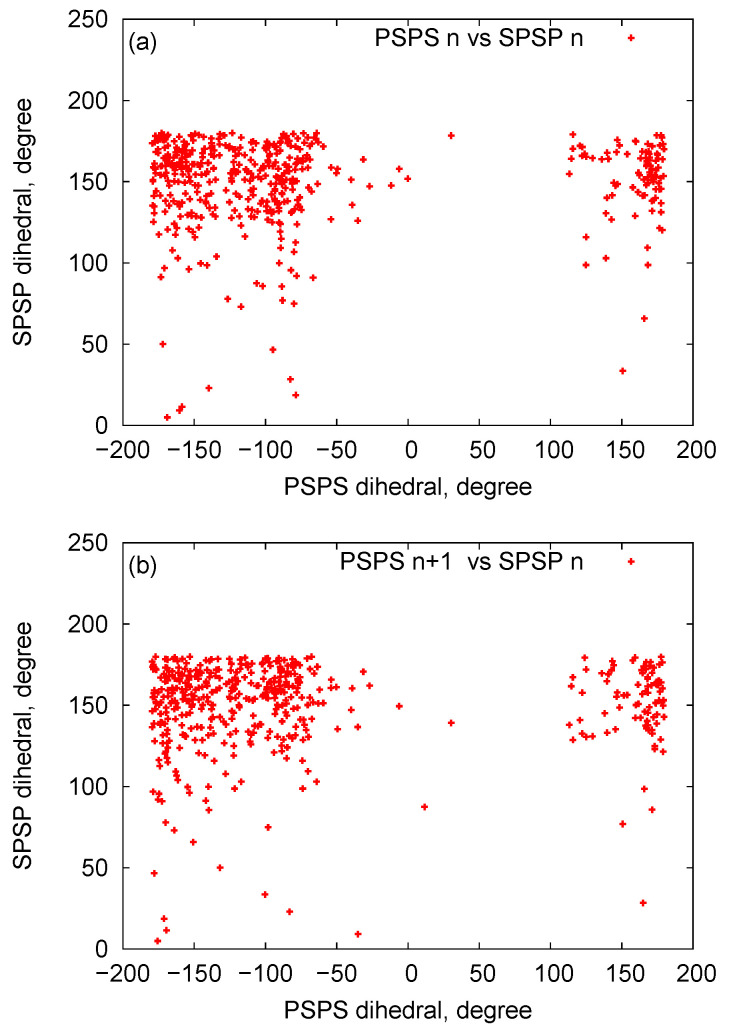
The two-dimensional map of the dihedral pair distributions of the RNA nanotube with two rings (**a**) Dihedral pairs centered around phosphate (**b**) dihedral pairs centered around sugar.

In Figure 8, we plot bead distances for P-C4 (phosphate—sugar ring) and C4-B (sugar ring—nucleobase) of two-ring RNA nanotube in the three-bead approximation of the coarse-grained model. In this plot, for a particular structure, the distances of the beads are plotted as a function of the residue index along the RNA strands of RNA nanotubes. It is clear that the distances remain almost constant with some fluctuations due to the damping of the system. Furthermore, in order to describe the three-bead coarse-grained structure, we have presented the two-dimensional maps for the dihedral pair distributions of two-ring RNA nanotube in Figure 9. These plots are similar to the η−θ plots described in Ref. [56]. The RNA conformation classes are described based on the two-dimensional plots of dihedral angle pair distributions. The conformation classes, that were separable from the majority of other such classes, corresponded to the kissing loop as mentioned in reference [28]. In the case of the two-ring nanotube, we see that the numbers of points in the two-dimensional plots were differently distributed in the plane. Some points in the plane were densely populated, whereas some were sparsely populated. The densely populated region of the plot corresponded to the area of interest because it possesseed significant classes of RNA conformations. In the two-dimensional plot for the two-ring RNA nanotube, we found that the maximally dense population of the points was around (165, 150). Furthermore, we found that the two-dimensional plots of dihedral angle pair distributions for the four-, five-, and 10-ring RNA nanotubes were in the vicinity of the same region for the two-ring RNA nanotube.

## 5. Conclusions

In summary, we have developed new coarse-grained models for different sized RNA nanotubes. Based on the three-bead approximation approach, we have used the center of mass of each bead to be one atom in the sugar ring, phosphate backbone, and the nucleobase, respectively. Based on the developed coarse-grained models, for multiple-ring RNA nanotubes, we have calculated the radial distribution functions, root mean square deviations, histograms for the dihedral angles of the backbone, and the angles of three beads. We have shown that the intensity of the radial distribution functions decreases with the increasing number of RNA nanorings in the RNA nanotube. We have also shown that there are significant classes of RNA conformations and that the peaks in the two-dimensional map of the dihedral pair distributions would be corresponding to the kissing loops. Finally, a number of physical properties and characteristics (e.g., radial distribution functions, angle and dihedral histograms, root mean square deviations, and others) of different size RNA nanotubes possess similar behaviors. This reveals that it is possible to efficiently manipulate such properties and characteristics for larger size RNA nanotubes, which are practically important for drug delivery and other biomedical applications of these structures. The developed models could be further generalized to account for more complex multiscale interactions, integrating our developed methodology with predictive, dynamic, and stochastic coarse-grained approaches [11,57,58,59,60,61,62]. This would allow us to better face challenges of a vast separation of spatial and temporal scales between processes happening at the atomic and cellular levels and to provide a route for more efficient integrations of atomistic and molecular information into larger-scale models.
